# Association between neutrophil percentage-to-albumin ratio and 3-month functional outcome in acute ischemic stroke patients with reperfusion therapy

**DOI:** 10.3389/fneur.2022.898226

**Published:** 2022-09-13

**Authors:** Ting Cui, Changyi Wang, Qiange Zhu, Shucheng Li, Yuan Yang, Anmo Wang, Xuening Zhang, Wenzuo Shang, Bo Wu

**Affiliations:** ^1^Center of Cerebrovascular Diseases, Department of Neurology, West China Hospital, Sichuan University, Chengdu, China; ^2^Department of Rehabilitation Medicine Center, West China Hospital, Sichuan University, Chengdu, China; ^3^The Second Department of Neurology, Shanxi Provincial People's Hospital, Xi'an, China

**Keywords:** acute ischemic stroke, reperfusion therapy, neutrophil, albumin, outcome

## Abstract

**Background:**

Neutrophils and albumin are associated with outcomes in patients with acute ischemic stroke (AIS). We aimed to explore the association between the neutrophil percentage-to-albumin ratio (NPAR), a novel marker of inflammation and oxidative stress, and the 3-month functional outcome in AIS patients with reperfusion therapy.

**Methods:**

This single-center, retrospective cohort study consecutively enrolled AIS patients with reperfusion therapy. Neutrophils and albumin were collected on admission. The primary outcome was a poor functional outcome, which was defined as a modified Rankin scale score of 3–6 at 3 months.

**Results:**

A total of 647 patients with AIS who received reperfusion therapy were analyzed. The mean age was 68.9 ± 13.9 years, and 358 (55.3%) of the patients were men. The median NPAR was 1.89 (interquartile range [IQR] 1.64–2.09). The percentage of patients with a 3-month poor functional outcome was 57.0% (369/647). NPAR was positively associated with a poor functional outcome (odds ratio [*OR*] 2.76, 95% *CI*: 1.52–5.03, *p* = 0.001). When patients were classified into tertiles, patients in the upper tertile (2.03–7.59) had a higher risk of poor outcome than patients in the lower tertile after adjusting for potential confounders (0.78–1.73) (*OR* 2.10, 95% *CI*: 1.28–3.42, *p* = 0.003). The risk of poor outcome increased with NPAR tertiles (*p*-trend = 0.003). The optimal cut-off value of the NPAR for predicting a poor outcome was 1.72, with a sensitivity of 0.75, and a specificity of 0.43.

**Conclusion:**

Neutrophil percentage-to-albumin ratio was significantly associated with 3-month poor functional outcomes in patients with AIS who received reperfusion therapy.

## Introduction

Reperfusion therapy has become the standard of care for patients with acute ischemic stroke (AIS) (1). However, approximately half of the patients with AIS suffer poor clinical outcomes after reperfusion therapy (2, 3). Inflammation and oxidative stress are two critical variables influencing the prognosis of patients with AIS after reperfusion therapy (4).

During the acute phase of AIS, neutrophils are the earliest inflammatory cells that are abundantly present in cerebral microvessels, and their subsequent release of reactive oxygen species (ROS) is thought to be the main cause of reperfusion injury after AIS (5–9). Some studies found that serum albumin played a key role in scavenging ROS (10, 11) and might exert an anti-inflammatory effect by inhibiting neutrophil spreading (12, 13). Neutrophils and albumin are associated with outcomes in patients with AIS (14–18).

The neutrophil percentage-to-albumin ratio (NPAR) is an emerging marker of inflammation and oxidative stress. The NPAR has been reported to have prognostic significance in patients with cancer, spinal cord injury, acute kidney injury, acute myocardial infarction, and cardiogenic shock (19–27). Recently, a retrospective study explored the association between NPAR and infection in patients with AIS (28). However, there is uncertainty regarding the association between NPAR and 3-month functional outcomes in AIS patients with reperfusion therapy. We hypothesized that NPAR may reflect the severity of inflammation and ROS damage in the acute phase of AIS. We sought to assess the association between NPAR and patient outcomes after reperfusion therapy.

## Materials and methods

### Patients

This retrospective cohort study consecutively enrolled patients with AIS admitted to West China Hospital between 1 January 2018 and 31 December 2020. Patients who received reperfusion therapies, such as intravenous thrombolysis and/or mechanical or thrombus aspiration thrombectomy (or both), were included. The exclusion criteria were as follows: (1) without measuring neutrophil or albumin within 24 h of stroke onset. (2) Patients with severe liver damage (alanine aminotransferase [ALT] ≥150 IU/L and/or aspartate aminotransferase [AST] ≥120 IU/L for men; alanine aminotransferase [ALT] ≥120 IU/L and/or aspartate aminotransferase [AST] ≥105 IU/L for women). (3) Patients with severe kidney damage (estimated glomerular filtration rate [eGFR] ≤15 ml/min/1.73 m^2^) (29). (4) Patients who failed to follow-up. This study was approved by the Scientific Research Department of West China Hospital. We obtained oral informed consent from each patient or their relative.

### Data collection

The variables collected included demographics (age and gender), medical histories (hypertension, atrial fibrillation, diabetes, hyperlipidemia, and coronary heart diseases), the National Institute of Health Stroke Scale (NIHSS) score, the Alberta Stroke Program Early CT Score (ASPECTS), hemorrhagic transformation (30), reperfusion therapy method, and the Trial of Org 10172 in Acute Stroke Treatment (TOAST) classification (31). Successful recanalization after endovascular therapy was defined as a modified Thrombolysis in Cerebral Infarction grading system score of 2b or 3 (32). Blood samples were collected for laboratory measurements in the emergency department. White blood cell (WBC) counts, including absolute numbers of neutrophils were measured using an automated hematology analyzer (Sysmex, Kobe, Japan). Serum albumin levels were determined with an Olympus AU-5400 automatic analyzer (Olympus, Tokyo, Japan).

### Outcome

Follow-up for all included patients was conducted by telephone at 3 months to evaluate their functional outcome. A 3-month modified Rankin scale score of ≥3 was regarded as a poor functional outcome (33).

### Statistical analysis

Means ± standard deviations (SDs) or medians (interquartile range [IQR]) were used to describe the quantitative variables as appropriate. Qualitative variables were reported as numbers and percentages. Student's *t*-test or the Mann–Whitney *U*-test for continuous variables and the χ^2^ test or Fisher's exact test for categorical data were used to conduct descriptive analyses of baseline characteristics and 3-month functional outcomes as appropriate. Variables within *p* < 0.10 in univariable analysis were defined as potential confounders. Multivariable logistic regression analysis was performed to explore the association between the NPAR and poor outcomes. Trends for the odds ratios (*OR*s) of poor outcome across NPAR tertiles (*p*-trend) were tested by entering the median value of NPAR in each tertile as a continuous variable (34). A receiver operating characteristic (ROC) curve was calculated to assess the diagnostic value of the NPAR in predicting outcome. The discrimination of NPAR was evaluated by the area under the ROC curve (AUC). The optimum NPAR cutoff point was determined by the Youden index. In addition, we compared the AUC of the NPAR with that of albumin, neutrophil percentage, and neutrophil-to-lymphocyte ratio in predicting a 3-month poor functional outcome (the Delong method) (35).

All analyses were performed using IBM SPSS Statistics (25.0; IBM, Armonk, NY, USA), R version 4.0.2 (R Foundation for Statistical Computing, Vienna, Austria), and MedCalc 15.2.2 (MedCalc Software bvba, Ostend, West Flanders, Belgium). A two-sided *p* value less than 0.05 was considered statistically significant.

## Results

### Baseline characteristics

**Figure 1:**
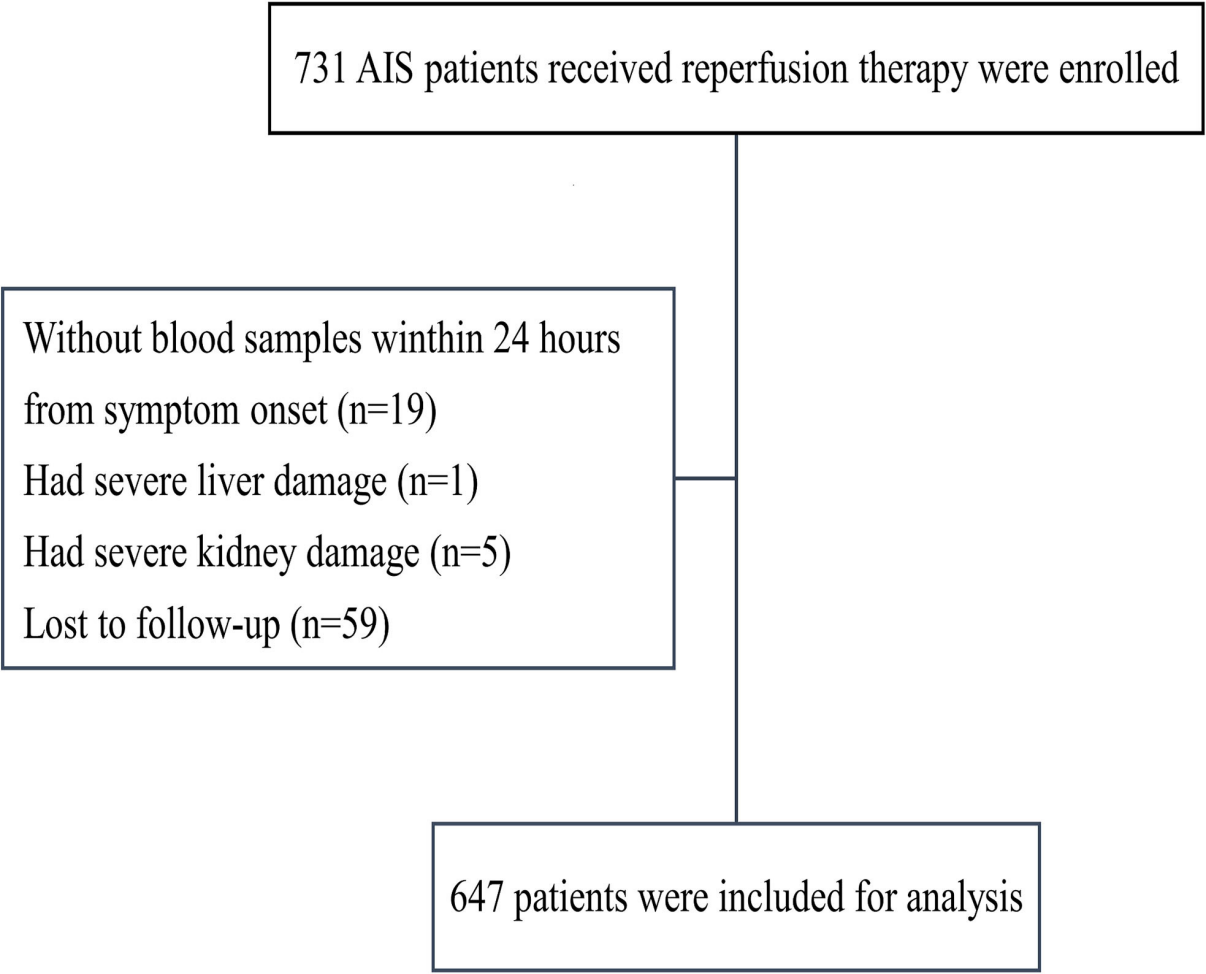
A flowchart of patient selection.

We enrolled 731 patients with reperfusion therapy; 19 patients were excluded because they did not have blood samples within 24 h of symptom onset; 6 patients had severe liver or kidney damage; and 59 patients were lost to follow-up (Figure 1). In total, 647 patients were included in this study.

As shown in Table 1, the mean age was 68.9 ± 13.9 years and 358 (55.3%) patients were men. The median NPAR was 1.89 (IQR 1.64–2.09). The median interval between stroke onset and blood sample measurement was 3.3 h (IQR 2.3–4.6 h). Approximately 32.0% (206/647) of patients received alteplase, 46% (297/647) of patients received endovascular therapy, and 22% (144/647) of patients received alteplase combined with endovascular therapy. The percentage of patients with a 3-month poor functional outcome was 57.0% (369/647). The baseline characteristics of patients stratified by the reperfusion therapy method are shown in Supplementary Table S1.

**Table 1:** Patient characteristics stratified by 3-month functional outcome.

**Variables**	**Overall**	**Good outcome**	**Poor outcome**	***P* value**
	**(*n =* 647)**	**(*n =* 278)**	**(*n =* 369)**	
Age, years, mean (SD)	68.9 (13.9)	64.8 (14.1)	72.0 (12.9)	<0.001
Male, *n* (%)	358 (55.3)	180 (64.7)	178 (48.2)	<0.001
Hypertension, *n* (%)	356 (55.0)	144 (51.8)	212 (57.5)	0.177
Diabetes, *n* (%)	149 (23.0)	55 (19.8)	94 (25.5)	0.108
Hyperlipemia, *n* (%)	54 (8.3)	26 (9.4)	28 (7.6)	0.509
Atrial fibrillation, *n* (%)	298 (46.1)	95 (34.2)	203 (55.0)	<0.001
Coronary heart diseases, *n* (%)	101 (15.6)	34 (12.2)	67 (18.2)	0.052
Current smoking, *n* (%)	166 (25.7)	92 (33.1)	74 (20.1)	<0.001
Alcohol consumption, *n* (%)	149 (23.0)	83 (29.9)	66 (17.9)	<0.001
Baseline NIHSS, median (Q1-Q3)	14 (9–18)	9 (5–14)	16 (12–20)	<0.001
^†^Baseline ASPECT, median (Q1-Q3)	8 (7–9)	9 (8–10)	8(7–9)	<0.001
White blood cell, *10^9^/L, mean (SD)	8.72 (3.48)	8.35 (2.89)	9.01 (3.84)	0.017
Albumin, g/L, mean (SD)	40.7 (4.0)	41.4 (3.6)	40.2 (4.2)	<0.001
Neutrophil, *10^9^/L, mean (SD)	6.79 (3.44)	6.32 (2.96)	7.15 (3.72)	0.002
Neutrophil percentage, median (Q1–Q3)	77.74 (67.71–85.34)	75.14 (64.23–83.88)	79.03 (70.29–86.10)	<0.001
NPAR, median (Q1–Q3)	1.89 (1.64–2.09)	1.81 (1.55–2.04)	1.94 (1.72–2.12)	<0.001
Serum glucose, mmol/L, mean (SD)	8.32 (2.99)	7.88 (2.80)	8.65 (3.09)	0.001
TOAST classification, n (%)				<0.001
Large-artery Atherosclerosis	209 (32.3)	96 (34.5)	113 (30.6)	
Cardio-embolism	*282(43.6)*	93 (33.5)	189 (51.2)	
Lacunar	*40 (6.2)*	34 (12.2)	6 (1.6)	
Other	*36 (5.6)*	18 (6.5)	18 (4.9)	
Undetermined	*80 (12.4)*	37 (13.3)	43 (11.7)	
Reperfusion therapy method, *n* (%)				<0.001
Thrombolysis only	*206 (31.8)*	116 (41.7)	90 (24.4)	
Thrombectomy only	297 (45.9)	99 (35.6)	198 (53.7)	
Thrombolysis and thrombectomy	*144 (22.3)*	63 (22.7)	81 (22.0)	
Interval between stroke onset and emergency department, h, median (Q1–Q3)	2.9 (2.0–4.0)	2.8 (2.0–3.6)	3.0 (1.9–4.0)	0.666
Interval between stroke onset and blood sample measurement, h, median (Q1–Q3)	*3.3 (2.3–4.6)*	3.2 (2.2–4.4)	3.4 (2.3–4.7)	0.241
^#^Hemorrhagic transformation, *n* (%)	*151 (24.4)*	*34 (12.4)*	*117 (34.0)*	<0.001
*Successful reperfusion, *n* (%)	*392 (88.9)*	*154 (95.1)*	*238 (85.3)*	0.003

^*^A total of 441 patients who received thrombectomy therapy were analyzed.

^#^A total of 619 patients who had follow-up non-contrast-enhanced computed tomography were analyzed.

^†^A total of 535 patients who had baseline ASPECT scores were analyzed. A total of 112 patients had missing ASPECT scores due to posterior circulation occlusion (*n* = 88), anterior cerebral artery occlusion (*n* = 7), missing baseline NCCT (*n* = 10), and poor image quality (*n* = 7).

SD, standard deviation; NIHSS, National Institutes of Health Stroke Scale; TOAST, the Trial of Org 10172 in Acute Stroke Treatment. NPAR, neutrophil percentage-to-albumin ratio.

### Association between NPAR and poor outcome

The univariable analysis showed that age, gender, baseline NIHSS score, atrial fibrillation, diabetes, coronary heart diseases, current smoking, drinking consumption, TOAST classification, serum glucose, reperfusion therapy method, and interval between stroke onset and blood sample measurement were significant confounders (*P* < 0.10, Table 2).

**Table 2:** Univariable logistic regression analysis of variables associated with poor 3-month outcome.

**Variable**	**Unadjusted odds ratio (95% confidence interval)**	***p*- value**
Age	1.04 (1.03, 1.05)	<0.001
Male	0.51 (0.37, 0.70)	<0.001
Hypertension	1.26 (0.92, 1.72)	0.153
Diabetes	1.39 (0.95, 2.02)	0.090
Hyperlipemia	0.80 (0.46, 1.39)	0.423
Atrial fibrillation	2.36 (1.71, 3.25)	<0.001
Coronary heart diseases	1.59 (1.02, 2.49)	0.041
Current smoking	0.51 (0.36, 0.73)	<0.001
Alcohol consumption	0.51 (0.35, 0.74)	<0.001
Baseline NIHSS score	1.18 (1.14, 1.22)	<0.001
Serum glucose	1.10 (1.04, 1.17)	0.001
Interval between stroke onset and emergency department	1.01 (0.95, 1.08)	0.654
Interval between stroke onset and blood sample measurement	1.04 (0.99, 1.09)	0.094
TOAST classification		
Large-artery Atherosclerosis	Reference	
Cardio-embolism	1.73 (1.20, 2.50)	0.004
Lacunar	0.15 (0.06, 0.37)	<0.001
Other	0.85 (0.42, 1.72)	0.652
Undetermined	0.99 (0.59, 1.66)	0.961
Reperfusion therapy method		
Thrombolysis only	Reference	
Thrombectomy only	2.58 (1.79, 3.72)	
Thrombolysis and thrombectomy	1.66 (1.08, 2.55)	0.021
*Successful reperfusion	0.30 (0.14, 0.66)	0.003
^#^Hemorrhagic transformation	3.65 (2.39, 5.58)	<0.001
^†^Baseline ASPECT score	0.75 (0.67, 0.84)	<0.001

NIHSS, National Institutes of Health Stroke Scale; TOAST, the Trial of Org 10172 in Acute Stroke Treatment.

^*^A total of 441 patients who received thrombectomy therapy were analyzed.

^#^A total of 619 patients who had follow-up non-contrast-enhanced computed tomography were analyzed.

^†^A total of 535 patients who had baseline ASPECT scores were analyzed. A total of 112 patients had missing ASPECT scores due to posterior circulation occlusion (*n* = 88), anterior cerebral artery occlusion (*n* = 7), missing baseline NCCT (*n* = 10), and poor image quality (*n* = 7).

In univariable analysis, the NPAR was significantly associated with a 3-month poor functional outcome when it was treated as a continuous variable (*OR* 3.44, 95% *CI*: 2.13–5.54, *p* < 0.001, Table 3). After multivariable adjustment, the association between NPAR and poor outcome remained significant (*OR* 2.76, 95% *CI*: 1.52–5.03, *p* = 0.001).

**Table 3:** Multivariate logistic regression analysis between admission NPAR and poor outcome^*^.

**Variable**	**Non-adjusted model**	**Adjusted model**
NPAR	3.44 (2.13, 5.54), <0.001	2.76 (1.52, 5.03), 0.001
NPAR tertiles	
T1(0.78–1.73)	Reference	Reference
T2(1.74–2.03)	1.85 (1.27, 2.72), 0.002	1.39 (0.87, 2.22), 0.166
T3(2.03–7.59)	2.54 (1.72, 3.75), <0.001	2.10 (1.28, 3.42), 0.003
*P*-trend	<0.001	0.003

^*^Results for each model are presented as odds ratios (95% confidence interval), p values.

Adjusted model: adjusted for age, gender, atrial fibrillation, diabetes, coronary heart diseases, current smoking, drinking consumption, baseline NIHSS score, serum glucose, interval between stroke onset and blood sample measurement, TOAST classification, and reperfusion therapy method.

NIHSS, National Institutes of Health Stroke Scale; TOAST, the Trial of Org 10172 in Acute Stroke Treatment.

When the NPAR was classified into tertiles, the *OR* of poor outcome was 1.85 for T2 and 2.54 for T3, when compared with T1 without adjustment. After multivariable adjustment, T3 still had a significant higher risk of poor outcome than T1 (*OR* 2.10, 95% *CI*: 1.28–3.42, *p* = 0.003), while T2 was no longer significantly different from T1 (*OR* 1.39, 95% *CI*: 0.87–2.22, *p* = 0.166). The risk of poor outcome significantly increased stepwise across NPAR tertiles (*p*-trend = 0.003).

The other four models were constructed to further explore the association between NPAR and outcome. In models 2, 3, and 4, after further adjustment for the ASPECT score, hemorrhagic transformation, and reperfusion status, respectively, the association between NPAR and outcome remained significant. In model 5, in addition to the variables in model 1, we further adjusted for the ASPECT score, hemorrhagic transformation, and reperfusion status together, and the association remained significant (*OR* 2.72, 95% *CI*: 1.19–6.26, *p* = 0.018, Supplementary Table S2).

### Predictive value of NPAR

**Figure 2:**
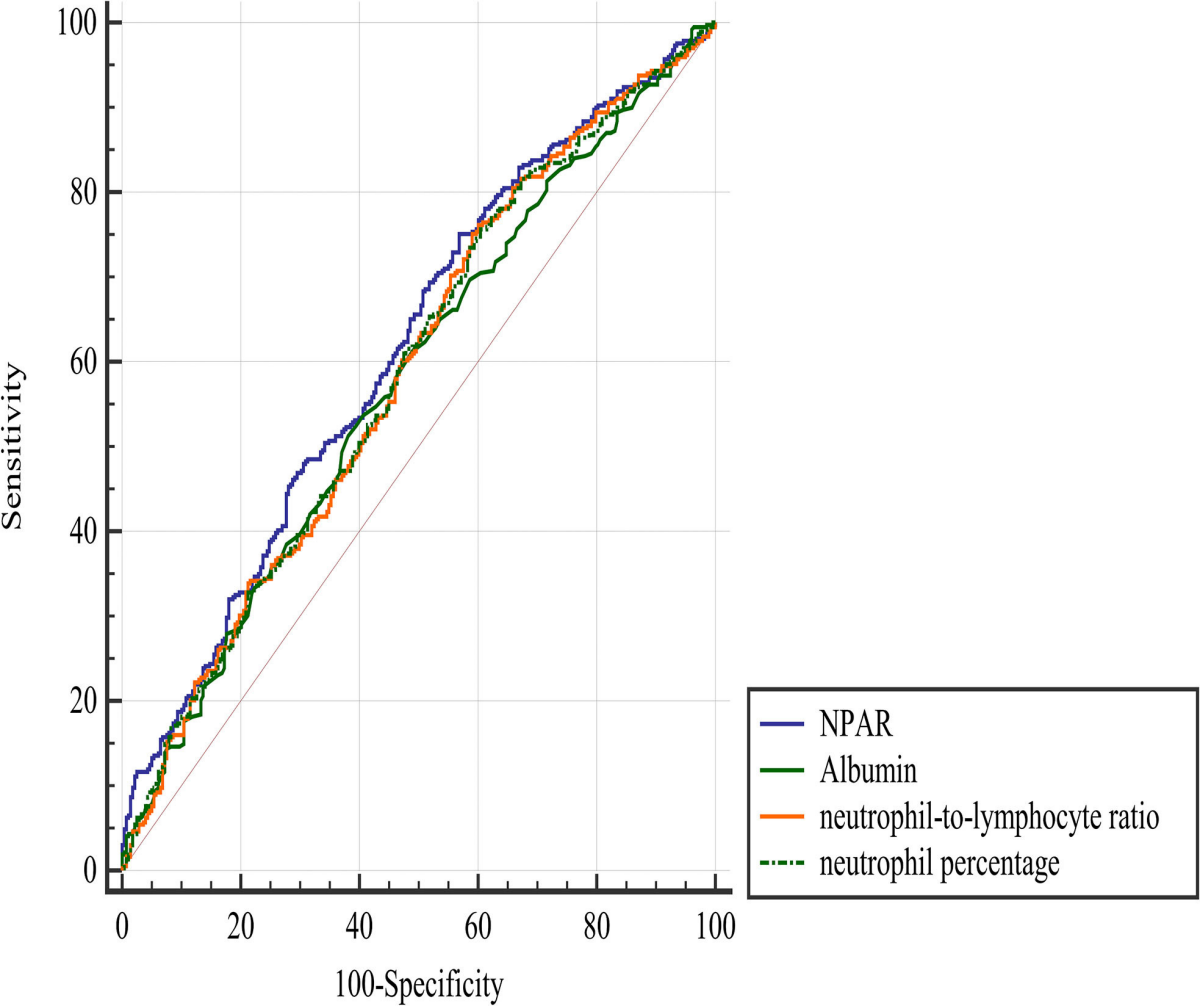
Comparison of predictive value between NPAR and other parameters in the prediction of a 3-month poor functional outcome. NPAR, neutrophil percentage-to-albumin ratio.

The ROC analysis suggested that the optimal cut-off value of the NPAR for predicting the 3-month poor functional outcome was 1.72, with a sensitivity of 0.75 and a specificity of 0.43 (Figure 2). Comparing the predictive value with other biomarkers, NPAR showed the highest AUC value [0.614 (0.575–0.652)] compared with albumin [0.578 (0.539–0.617)], neutrophil percentage [0.588 (0.549–0.626)], and neutrophil-to-lymphocyte ratio [0.588 (0.549–0.626)], but there was no significant difference between NPAR and albumin (*p* = 0.144).

## Discussion

In this retrospective observational study, we investigated the association between NPAR and 3-month functional outcome in patients with AIS who were treated with reperfusion therapies. We found that NPAR was positively associated with poor outcomes. The optimal cutoff value of the NPAR for predicting a poor outcome was 1.72 with a sensitivity of 0.75 and a specificity of 0.43. The predictive value of NPAR was significantly higher than that of neutrophil percentage and neutrophil-to-lymphocyte ratio but not albumin.

Post-ischemic inflammation and oxidative stress play essential roles in the response to brain ischemia-reperfusion injury in patients with AIS after reperfusion therapy (36). As markers of inflammation and oxidative stress response, neutrophils and albumin have been found to be associated with outcomes in AIS patients with reperfusion therapy (14–18). NPAR, which combines neutrophils with albumin, is accessible in daily clinical practice. Monitoring NPAR may help clinicians detect patients at high risk of poor outcome. However, the association between NPAR and outcome in AIS patients with reperfusion therapies remains unclear. Recently, a retrospective study found that NPAR could predict the occurrence of stroke-associated infection, but it did not focus on patients with reperfusion therapy and did not have data on the short-term outcome of patients with AIS (28). In the current study, we found that NPAR was positively associated with the poor functional outcome, and that the predictive value of NPAR for outcome was significantly higher than that of other conventional biomarkers, for example, the neutrophil-to-lymphocyte ratio, in these patients.

The underlying mechanism of the association between NPAR and outcome remains unclear and could be explained as follows: on one hand, during the acute phase of AIS, numerous pro-inflammatory cytokines and damage-associated molecular patterns (DAMPs) are released that promote neutrophil recruitment and activation, which in turn promote the release of ROS and ultimately result in poor functional outcome (5–9). On the other hand, it has been found that albumin may help limit the production of ROS and further scavenge ROS (10, 12). In addition, some studies found that albumin can also exert an anti-inflammatory effect by inhibiting neutrophil spreading (12, 13).

Our study has some limitations. First, this was a single-center retrospective study, which could lead to selection bias. Second, the interval between symptom onset and admission measurement of blood samples might lead to bias. However, we adjusted for this variable in multivariable analysis, and the result was significant. Third, regarding AUC, the cut-off value of its sensitivity and specificity was fairly low, and the clinical significance of NPAR may be limited. Fourth, although we adjusted for potential confounders using multivariate models, neutrophils may be influenced by infectious diseases, hematological or rheumatic disorders, and a history of long-term immunosuppressant drug use. Due to the retrospective study design, we could not adjust for these variables in our study. Fifth, we could not explore the association between NPAR and early neurological deterioration in this study due to limited data. Finally, previous studies have found that GFAP and S100B correlated with stroke severity and outcome (37, 38). However, due to the retrospective study design, we could not provide data on these prognostic biomarkers in our center, therefore, we could not compare the advantages and disadvantages between NPAR and these biological markers.

## Conclusions

Neutrophil percentage-to-albumin ratio was independently associated with a 3-month poor functional outcome in AIS patients with reperfusion therapy.

## Data availability statement

The data that support the findings of this study are available from the corresponding author upon reasonable request.

## Ethics statement

The studies involving human participants were reviewed and approved by the Scientific Research Department of West China Hospital. Written informed consent for participation was not required for this study in accordance with the national legislation and the institutional requirements.

## Author contributions

BW conceived and designed the study. TC, CW, QZ, AW, XZ, SL, YY, and WS acquired the data, which TC analyzed. TC and CW aided in data interpretation and wrote the manuscript. All authors were involved in revising the article and approved the final version.

## Funding

This work was supported by the National Natural Science Foundation of China (82071320 and 81870937), and the 1.3.5 project for disciplines of excellence, West China Hospital, Sichuan University (ZYGD18009).

## Conflict of interest

The authors declare that the research was conducted in the absence of any commercial or financial relationships that could be construed as a potential conflict of interest.

## Publisher's note

All claims expressed in this article are solely those of the authors and do not necessarily represent those of their affiliated organizations, or those of the publisher, the editors and the reviewers. Any product that may be evaluated in this article, or claim that may be made by its manufacturer, is not guaranteed or endorsed by the publisher.
