# Supplementary material for: Association between neutrophil percentage-to-albumin ratio and 3-month functional outcome in acute ischemic stroke patients with reperfusion therapy
Source: Front Neurol. 2022 Sep 13;13:898226. doi: 10.3389/fneur.2022.898226 (PMC9513151; doi:10.3389/fneur.2022.898226)
Supplement: Supplementary file 1 [file Table_1.DOCX]

Table S1. Patient characteristics stratified by reperfusion therapy method

| Variables | thrombolysis only | thrombectomy only | thrombolysis and  thrombectomy | P value |
| --- | --- | --- | --- | --- |
|  | (n=206) | (n=297) | (n=144) |  |
| Age, years, mean (SD) | 70.2 (13.4) | 68.7 (13.8) | 67.5 (14.7) | 0.174 |
| Male, n (%) | 113 (54.9) | 162 (54.5) | 83 (57.6) | 0.817 |
| Hypertension, n (%) | 122 (59.2) | 152 (51.2) | 82 (56.9) | 0.178 |
| Diabetes, n (%) | 56 (27.2) | 61 (20.5) | 32 (22.2) | 0.212 |
| Hyperlipemia, n (%) | 24 (11.7) | 17 (5.7) | 13 (9.0) | 0.058 |
| Atrial fibrillation, n (%) | 73 (35.4) | 151 (50.8) | 74 (51.4) | 0.001 |
| Coronary heart diseases, n (%) | 28 (13.6) | 52 (17.5) | 21 (14.6) | 0.457 |
| Current smoking, n (%) | 53 (25.7) | 76 (25.6) | 37 (25.7) | 0.999 |
| Alcohol consumption, n (%) | 46 (22.3) | 67 (22.6) | 36 (25.0) | 0.815 |
| Baseline NIHSS, median (Q1-Q3)  *ASPECT Score, median (Q1-Q3) | 9 (4-14)  9 (7-10) | 15 (12-20)  8 (7-9) | 14 (11-18)  8 (7-9) | <0.001  0.001 |
| White blood cell, *10^9/L, mean (SD) | 8.19 (3.11) | 8.97 (3.77) | 8.96 (3.27) | 0.03 |
| Albumin, g/L, mean (SD) | 41.3 (3.6) | 40.0 (4.4) | 41.5 (3.5) | <0.001 |
| Neutrophil, *10^9/L, mean (SD) | 6.05 (3.17) | 7.21 (3.60) | 6.99 (3.30) | 0.001 |
| NPAR, median (Q1-Q3) | 1.74 (1.53-2.01) | 1.97 (1.76-2.15) | 1.89 (1.63-2.07) | <0.001 |
| Serum glucose, mmol/L, mean (SD) | 8.29 (3.02) | 8.28 (3.04) | 8.42 (2.86) | 0.886 |
| TOAST classification, n (%) |  |  |  | <0.001 |
| Large-artery Atherosclerosis | 67 (32.5) | 94 (31.6) | 48 (33.3) |  |
| Cardio-embolism | 64 (31.1) | 153 (51.5) | 65 (45.1) |  |
| Lacunar | 38 (18.4) | 1 (0.3) | 1 (0.7) |  |
| Other | 6 (2.9) | 20 (6.7) | 10 (6.9) |  |
| Undetermined | 31 (15.0) | 29 (9.8) | 20 (13.9) |  |
| ^#^Successful reperfusion, n (%)  ^†^Hemorrhagic transformation, n (%)  ECASS classification, n (%) | NA  25 (12.1) | 266 (89.6)  80 (29.3) | 126 (87.5)  46 (32.9) | 0.628  <0.001  0.119 |
| HI-1 | 3 (8.1) | 14 (13.1) | 11 (20.4) |  |
| HI-2 | 6 (16.2) | 29 (27.1) | 14 (25.9) |  |
| PH-1  PH-2 | 1 (2.7)  15 (40.5) | 13 (12.1)  24 (22.4) | 6 (11.1)  15 (27.8) |  |
| Interval between stroke onset and emergency department, h, median (Q1-Q3) | 2.0 (1.5-3.0) | 3.0 (2.0-4.0) | 2.5 (1.9-3.4) | <0.001 |
| Interval between stroke onset and blood sample measurement, h, median (Q1-Q3)  Poor outcome, n (%) | 2.9 (2.1-3.7)  90 (43.7) | 3.9 (2.6-5.0)  198 (66.7) | 3.1 (2.2-4.2)  81 (56.2) | <0.001  <0.001 |

^*^A total of 535 patients who had baseline ASPECT scores were analyzed.

^#^A total of 441 patients who received thrombectomy therapy were analyzed.

^†^A total of 619 patients who had follow up NCCT were analyzed.

SD, standard deviation; NIHSS, National Institutes of Health Stroke Scale; TOAST: the Trial of Org 10172 in Acute Stroke Treatment; NCCT, noncontrast-enhanced computed tomography; ECASS, European Cooperative Acute Stroke Study.
