# Supplementary material for: Association between neutrophil percentage-to-albumin ratio and 3-month functional outcome in acute ischemic stroke patients with reperfusion therapy
Source: Front Neurol. 2022 Sep 13;13:898226. doi: 10.3389/fneur.2022.898226 (PMC9513151; doi:10.3389/fneur.2022.898226)
Supplement: Supplementary file 2 [file Table_2.DOCX]

Table S2. Multivariate logistic regression analysis between admission NPAR and poor outcome*

| Variable | NPAR |
| --- | --- |
| Nonadjusted model  Model 1  Model 2  Model 3  Model 4  Model 5 | 3.44 (2.13, 5.54), <0.001  2.76 (1.52, 5.03), 0.001  2.38 (1.26, 4.49), 0.008  2.73 (1.46, 5.10), 0.002  3.20 (1.52, 6.76), 0.002  2.72 (1.19, 6.26), 0.018 |

* Results for each model are presented as odds ratios (95% confidence intervals), p values.

Model 1: adjusted for age, gender, atrial fibrillation, diabetes, coronary heart diseases, current smoking, drinking consumption, baseline NIHSS score, serum glucose, interval between stroke onset and blood sample measurement, TOAST classification, and reperfusion therapy method.

Model 2: adjusted for variables in adjusted model 1 and ASPECT score.

Model 3: adjusted for variables in model 1 and hemorrhagic transformation.

Model 4: adjusted for variables in model 1 and successful reperfusion.

Model 5: adjusted for variables in adjusted model 1, ASPECT score, hemorrhagic transformation, and successful reperfusion.

NIHSS, National Institutes of Health Stroke Scale; TOAST: the Trial of Org 10172 in Acute Stroke Treatment.
